# 

**Published:** 1885-12-20

**Authors:** 


SUBSCRIBERS ON RENEWAL.—To any subscriber who, on renewal for 1886, will send us a new cash subscriber, the journal will be sent him at half-price; or for $3 we will send the journal to himself and his friend.

*RECEIPTED.*

For 1885—P. M. Catching, I. W. Bradley, to August; W. I. Brewington, E. W. Lane, I. W. Medlock, F. M. Fitzhugh, T. N. Skeen, J. T. Cleveland, G. R. Dozier, Z. T. Young, J. M. Lewis, T. M. Beatey, I. H. Henry' C. D. Tatman, M. R. Toland, Jno. A. Gordon, M. F. Alford.

For 1884—H. T. Shiel.

For 1883—G. G. Delbum.

For 1886—R. D. Jackson, A. W. Irwin, J. S. Carothers, S. J. Ellis, to April; W. M. Peacock, to March; W. I. McAlpine.

